# Documenting Trends in Malaria Data Reporting Accuracy Using Routine Data Quality Audits in Zambia, 2015–2022

**DOI:** 10.4269/ajtmh.24-0429

**Published:** 2024-11-26

**Authors:** Smita Das, Arantxa Roca Feltrer, Marie-Reine I. Rutagwera, Christopher Lungu, Prudence Malama, Mathews Monde, Ignatius Banda, Mercy M. Ingwe, Busiku Hamainza, Adam Bennett, Michael Hainsworth

**Affiliations:** ^1^ PATH Malaria Control and Elimination Partnership (MACEPA), Seattle, Washington;; ^2^ PATH MACEPA, Maputo, Mozambique;; ^3^ PATH MACEPA, Lusaka, Zambia;; ^4^ Zambia Ministry of Health, National Malaria Elimination Centre, Lusaka, Zambia

## Abstract

Since 2015, the Zambia National Malaria Elimination Centre has conducted routine data quality audits in Central, Southern, and Western provinces, but trends in data reporting accuracy have not been examined. Routine data quality audit data collected at health facilities reporting into the monthly health management information system (HMIS) and weekly malaria rapid reporting system (MRRS) were used to measure data reporting accuracy trends from 2015 to 2022 and potential influencing factors using three data elements: outpatient department attendance and rapid diagnostic test (RDT)-tested cases for HMIS and MRRS, total confirmed cases for HMIS only, and RDT-positive cases for MRRS only. Reporting accuracies for HMIS and MRRS data elements and the percentage of facilities reporting with high accuracy (≥85%) improved over this period. Low-accuracy (<70%) health facilities were uncommon, accounting for less than 15% of facilities for HMIS and MRRS. With each successive DQA visit, the proportion of facilities with high accuracy increased from visits 1 to 8: 23% to 56% (HMIS) and 42% to 85% (MRRS). No correlation was observed between facility size or incidence and overall accuracy for HMIS and MRRS. Starting in 2017, about 40–50% of health facilities appeared to be overreporting incidence in comparison with their register-based incidence. The risk stratification determined by register-based and reported incidences matched in more than 70% of facilities. Routine data quality audits conducted between 2015 and 2022 in Central, Southern, and Western provinces showed an improvement in malaria data reporting accuracy.

## INTRODUCTION

A robust surveillance system is the backbone of an effective malaria program. It is essential that the performance of a surveillance system is monitored through a set of metrics to ensure quality data, as measured by reporting rate, reporting completeness, timeliness, accuracy, consistency, and validity. Particularly for accuracy, data quality audits (DQAs) at the service delivery level are one way for country malaria programs to gauge the level of confidence they can have in reported data, as well as to identify strengths and weaknesses of the reporting process.

During DQAs, register and reported values are compared with measure data reporting accuracy across key malaria data elements. The results can reveal systematic errors in the transmission of malaria data from registers to each level of the reporting hierarchy, as well as provide an estimate of the level of underreporting or overreporting for certain malaria data indicators. Generally, for health facilities and community health workers (CHWs) that participate in routine DQAs (RQDAs), results are synthesized by district to develop individualized corrective action plans and prioritize data quality improvement activities. However, conducting RDQAs requires significant human and financial resources, which can limit the scope and scale-up. There is also no standard method for assessing temporal trends in aggregate data reporting accuracy using DQA results, highlighting a critical gap in programmatic decision-making using DQA results. Therefore, a systematic approach is urgently needed, not only to track trends in data reporting accuracy but also to establish DQA best practices tailored to evolving program needs so that limited resources are efficiently and effectively used.

Since 2015, the Zambia National Malaria Elimination Centre (NMEC) in partnership with the PATH Malaria Control and Elimination Partnership in Africa has conducted annual DQAs of malaria data reported into the monthly health management information system (HMIS) and the weekly malaria rapid reporting system (MRRS) at health facilities in Central, Southern, and Western provinces. Currently, health facility DQA results are used to measure monthly (HMIS) and weekly (MRRS) reporting accuracy, as well as to aggregate data reporting accuracy for 16 HMIS and 13 MRRS malaria data elements and overall. These data provide district-level officers with an annual snapshot of reporting accuracy at both district and health facility levels, but no further temporal analysis is done to assess accuracy trends. In this study, temporal trends in aggregate individual data element and overall malaria data reporting accuracies using 2015–2022 health facility HMIS and MRRS DQA results from Central, Southern, and Western provinces were characterized and used to inform recommendations for improving the DQA process and evaluation of accuracy trends.

## MATERIALS AND METHODS

### Malaria reporting in Zambia.

Routine malaria data in Zambia are collected in three surveillance systems: HMIS, MRRS, and Integrated Disease Surveillance Response. Since RQDAs occur only for data reported by health facilities in the HMIS and MRRS, this study focused on HMIS and MRRS DQA results. The malaria data flow process begins primarily at the health facility level for HMIS and at both health facility and community levels for MRRS (Supplemental Figure 1). At health facilities, staff record malaria data in patient and laboratory registers, which are then aggregated monthly (HMIS) or weekly (MRRS) using tally sheets before they are recorded into their respective reporting forms. The paper-based health facility reporting form for HMIS is submitted to the district health staff, who review and enter the data using a computer into the district health information system 2 (DHIS2). For MRRS, health facility staff enter the weekly aggregated malaria data directly into the MRRS DHIS2 by use of a mobile device.

### Study area.

**Figure 1.**
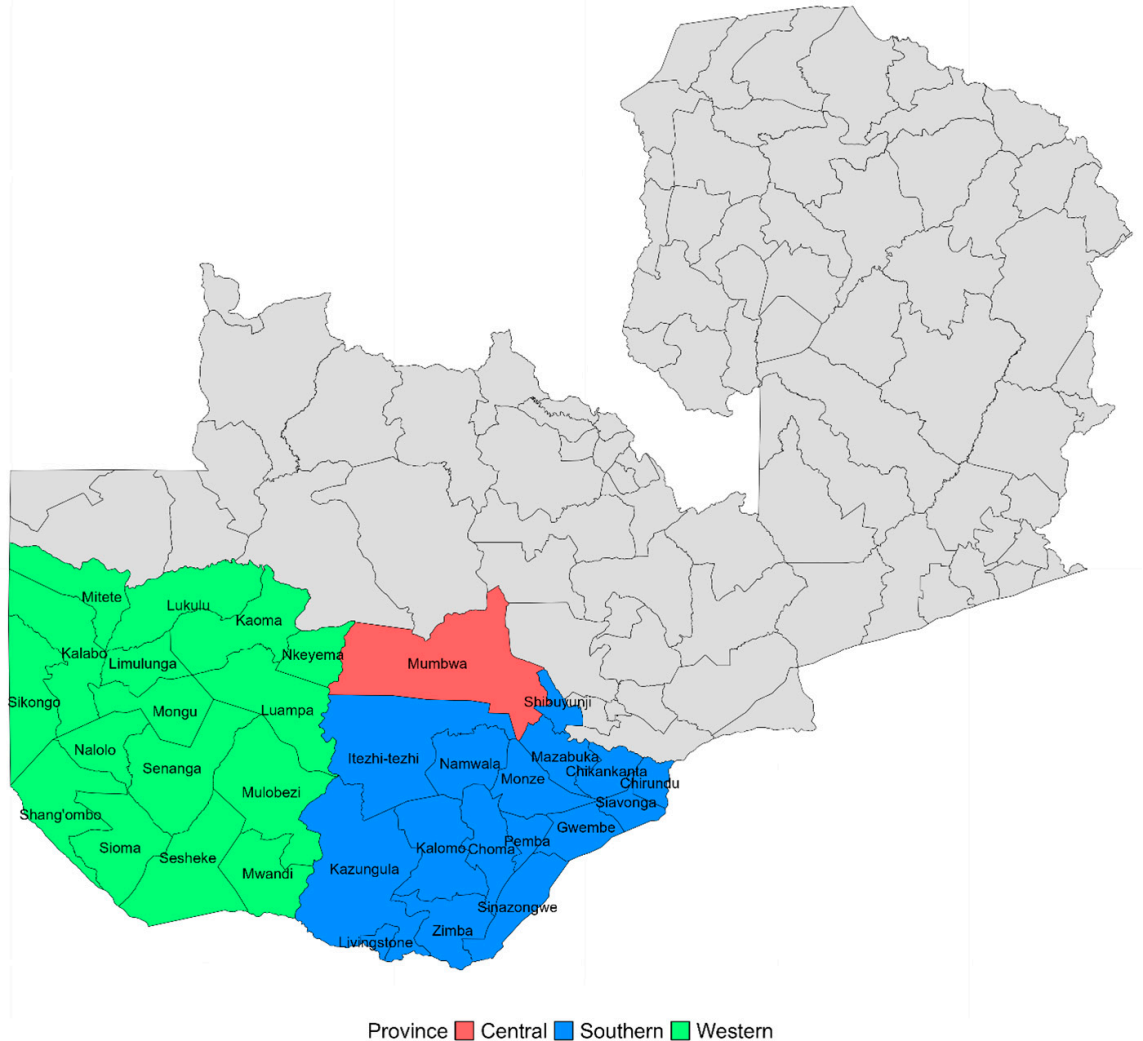
Districts that participated in routine data quality audits in Zambia from 2015 to 2022.

Routine data quality audit data for this study were collected from health facilities in Central, Southern, and Western provinces in Zambia (Figure 1). These three provinces are home to approximately six million people, or 31% of the total population.^1^ Malaria prevalence by rapid diagnostic test (RDT) varies across provinces: 22% in Central Province, 3% in Southern Province, and 47% in Western Province, with considerable variation within each province depending on local geological and environmental factors.^2^ There is generally higher transmission intensity in areas along Lake Kariba in Southern Province and near swamps, wetlands, and along the Zambezi River basin in Western Province.^3^ All other areas away from water bodies have low transmission.^3^ Annual stratification of health facility catchment areas based on malaria surveillance data is conducted by the NMEC and guides the use of specific interventions based on assignment to malaria risk strata.^4^

### RDQAs.

Routine data quality audits have been conducted annually since 2013. The approach and tools currently used for RDQAs, which are the subject of this study, were implemented starting in 2015. Routine data quality audits are done at 10–13 health facilities per district using purposive and random sampling methods. The criteria for site selection are all study sites (intervention and control), sites with low or inconsistent reporting, and sites that frequently violate data validation rules or consistently have poor data quality and include at least some high-performing sites.^5^ Every facility in a district is expected to be audited at least once every 2 years.

Routine HMIS and MRRS DQAs were introduced in Southern Province in 2015 across 13 and 14 districts, respectively, and then expanded to Central Province in 2016 (one district) and Western Province in 2017 (eight districts). By 2022, RDQAs were conducted in 33 districts, including two districts in Central Province, 15 districts in Southern Province, and 16 districts in Western Province; in total, 670 health facilities received at least one HMIS DQA, 678 health facilities received at least one MRRS DQA, and 662 health facilities received both HMIS and MRRS DQAs at least once.

During a DQA, one to two facilities in a district are audited per day, and 6 months of HMIS, MRRS, and CHW data are examined. This study focused specifically on HMIS and MRRS data only, primarily because of the inconsistent availability of CHW registers during audits. The 6-month audit typically encompasses the high transmission season each year, January to June. The district audit teams are typically composed of three district-level staff plus one or two additional staff from another district or province, NMEC or provincial staff, and any supporting project staff. Prior to conducting DQAs, audit teams participate in an orientation meeting and Excel-based DQA tools are prepopulated with data reported in HMIS and MRRS. Audit teams travel to the selected facilities and conduct audits onsite, where register data are entered into a laptop containing the prepopulated DQA tools to facilitate comparison between register and reported data. Data quality audit tool outputs are available in real time and are promptly shared at both the health facility and district levels, facilitating discussion about discrepancies, technical mentorship, and tailored action plans. At the district level, a review meeting is held to review the collective health facility DQA results and action plans. After all districts have completed DQAs, a province-level meeting is held to review and compare DQA results and data quality dashboards, debrief on the DQA experience, review health facility action plans, and further develop district action plans.

### Data reporting accuracy.

For this study, HMIS and MRRS DQA data from facilities in Central, Southern, and Western provinces during the 2015–2022 period were evaluated for trends in malaria data reporting accuracy by comparing reported values to corresponding register values. Three data elements were used to measure data reporting accuracy in the two systems: total outpatient department (OPD) attendance and RDT-tested cases for both HMIS and MRRS, total confirmed cases for HMIS, and RDT-positive cases for MRRS. These data elements were selected for their relevance to malaria indicators that are used routinely by NMEC for programmatic decision-making. If a monthly or weekly register value for a data element in the DQA tool was missing (blank), then that month or week was excluded from analysis. In the remaining monthly or weekly register and reported values, any missing (blank) reported values were updated to zero. For each data element, the weighted absolute percentage error (WAPE) method was applied to determine the aggregate percentage error between register values and reported values (HMIS or MRRS) for each health facility per audit year. To prevent a zero-sum denominator ($\sum_{i=1}^{n}y_{i})$ when the WAPE was applied, 1 was added to both the register and reported values for an individual month or week if the register value was zero. The WAPE was calculated as follows: $\mathrm{WAPE}=\;\frac{\sum_{i=1}^{n}\left|y_{i}-{\hat{y}}_{i}\right|}{\sum_{i=1}^{n}y_{i}}$ × 100, where *n* represents the number of months (HMIS) or weeks (MRRS), $y_{i}$ represents the register value, and ${\hat{y}}_{i}$ represents the reported (HMIS or MRRS) value.^6^ The aggregate individual data element reporting accuracy for each health facility per audit year was calculated by 100 − WAPE. The overall aggregate data reporting accuracy for each health facility per audit year was determined by the mean of its three data element aggregate accuracies. Per NMEC’s current practice, aggregate data reporting accuracy strata in this analysis were defined as high (≥85%), medium (≥70–85%), and low (<70%). Health facilities that received at least three DQA visits and had low overall aggregate data reporting accuracy (<70%) at more than half of the visits were considered recurrent-low-accuracy health facilities.

### Malaria case incidence.

The audit period incidence based on the registers and reporting systems for each health facility was calculated to characterize underreporting and overreporting. For each facility and audit period, the total confirmed cases and RDT-positive cases were aggregated from registers and each reporting system (HMIS and MRRS, respectively). To maximize the number of malaria cases captured by the two systems and to account for facilities where only one or the other reporting system was audited or to handle missing register and/or reported values in either of the reporting systems, the larger of the two data elements’ values from the registers and the larger of the two data elements’ values from the reporting systems were selected to generate a conservative estimate of the register-based incidence and reported incidence for each audit period. The register-based incidence and reported incidence were calculated using available population data from DHIS2 in Zambia and were reported per 1,000 population.

## STATISTICAL ANALYSES

Register and reporting system (HMIS and MRRS) data compiled from each audit’s DQA tool were processed using Microsoft Excel Power Query and analyzed using R statistical software. The distribution, average, median, and interquartile range (IQR) of health facility data element aggregate accuracies and overall aggregate data reporting accuracy from each reporting system (HMIS or MRRS) from 2015 to 2022 were characterized using box plots. The percentage of health facilities in each accuracy strata based on individual data element accuracies and overall data reporting accuracy across audit years was examined. Descriptive statistics were also applied to identify and describe data reporting accuracy trends at recurrent-low-accuracy health facilities. Additionally, the percentage of health facilities in each accuracy strata based on overall data reporting accuracy at each DQA visit 1–8 was assessed.

Pearson correlation analysis was performed to measure the strength of a linear correlation of 1) health facility size using total OPD attendance and overall data reporting accuracy, and 2) health facility register-based or reported incidence and overall data reporting accuracy for both HMIS and MRRS. Statistical significance was defined as a *P*-value ≤0.05. The register-based and reported incidences based on each audit period per health facility were compared to determine if the reported incidence was underreported (reported incidence < register-based incidence), overreported (reported incidence > register-based incidence), or the same as the register-based incidence. To assess the extent to which precision of the malaria incidence estimates affected annual risk stratification, an adjusted agreement metric was calculated; an arbitrary 10% allowable reporting error was explored, whereby the register-based incidence and reported incidence for a given health facility were considered the same if the reported incidence was within ±10% of the register-based incidence. The register-based and reported incidences, with and without adjustment, were then assigned to malaria risk strata and compared for agreement. The malaria risk strata were determined by current levels of malaria transmission outlined in Zambia’s *National Malaria Elimination Strategic Plan 2022–2026*: “high” level 4 is ≥500 cases per 1,000 population/year, “moderate” level 3 is between 200 and 499 cases per 1,000 population/year, “low” level 2 is between 50 and 199 cases per 1,000 population/year, “very low” level 1 is between 1 and 49 cases per 1,000 population/year, and “no malaria” level 0 is 0 cases per 1,000 population/year.^4^

## RESULTS

### Individual data elements’ aggregate data reporting accuracy.

#### HMIS.

**Figure 2.**
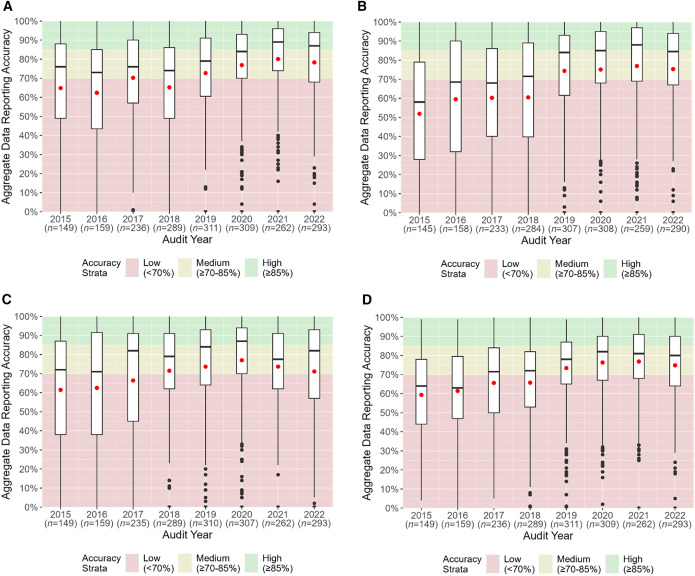
Aggregate data reporting accuracy using routine health management information system data quality audit results for total outpatient department (OPD) attendance (**A**), rapid diagnostic test (RDT)-tested cases (**B**), total confirmed cases (**C**), and overall (**D**).

From 2015 to 2022, the average health facility HMIS aggregate data reporting accuracy increased from 65% to 78% for total OPD attendance, from 52% to 75% for RDT-tested cases, and from 61% to 71% for total confirmed cases (Figure 2A–C; Supplemental Table 2). All three data elements had average data reporting accuracies that improved from low-accuracy to medium-accuracy strata during the assessment period. The median health facility HMIS data reporting accuracy also increased from 2015 to 2022: from 76% to 87% for total OPD attendance, from 58% to 85% for RDT-tested cases, and from 72% to 82% for total confirmed cases (Figure 2A–C; Supplemental Table 2). From 2015 to 2022, the median aggregate data reporting accuracy improved from medium- to high-accuracy strata for total OPD attendance and from low- to medium-accuracy strata for RDT-tested cases. The median aggregate data reporting accuracy for total confirmed cases shifted from medium-accuracy strata to high-accuracy strata from 2015 to 2020 and then returned to medium-accuracy strata by 2022. Interquartile ranges across all data elements decreased during the assessment period (Figure 2A–C; Supplemental Table 2).

#### MRRS.

**Figure 3.**
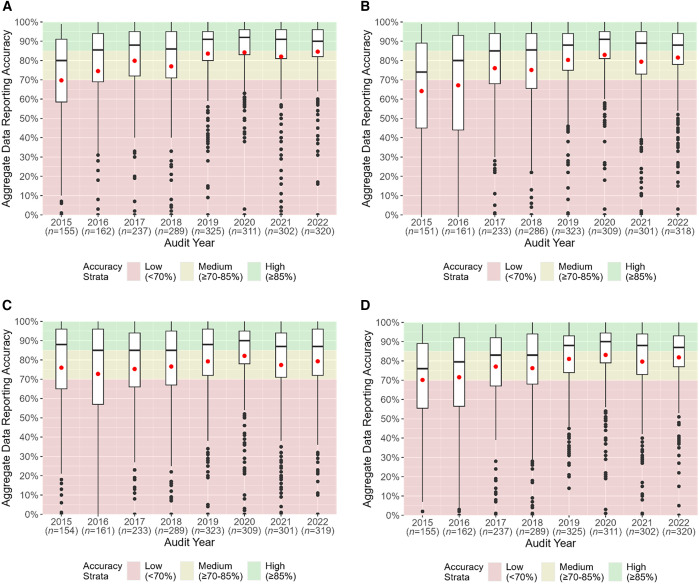
Aggregate data reporting accuracy using routine malaria rapid reporting system data quality audit results for total outpatient department (OPD) attendance (**A**), rapid diagnostic test (RDT)-tested cases (**B**), RDT-positive cases (**C**), and overall (**D**).

The average health facility MRRS aggregate data reporting accuracy for each data element increased from 2015 to 2022: from 70% to 85% for total OPD attendance, from 64% to 82% for RDT-tested cases, and from 76% to 79% for RDT-positive cases (Figure 3A–C; Supplemental Table 3). Of the three data elements, only the average reporting accuracy for RDT-tested cases improved from low- to medium-accuracy strata during the assessment period, whereas the average data reporting accuracy for total OPD attendance and RDT-positive cases remained in the medium-accuracy strata. Similarly, the median health facility MRRS aggregate data reporting accuracy for two data elements increased during the assessment period, from 80% to 90% for total OPD attendance and from 74% to 88% for RDT-tested cases, whereas RDT-positive cases decreased by one percentage point from 88% to 87% (Figure 3A–C; Supplemental Table 3). The median data reporting accuracies for total OPD attendance and RDT-tested cases improved from medium to high strata, and RDT-positive cases remained in the high strata. The IQR showed a gradual decrease from 2015 to 2022 (Figure 3A–C; Supplemental Table 3).

### Overall aggregate data reporting accuracy.

#### HMIS.

From 2015 to 2022, the average HMIS overall aggregate data reporting accuracy increased from 59% to 75% (Figure 2D; Supplemental Table 2). The median overall aggregate data reporting accuracy also increased from 64% to 80% during the assessment period (Figure 2D; Supplemental Table 2). Both the average and median overall data reporting accuracies shifted from low-accuracy strata in 2015 to medium-accuracy strata in 2022. Like the HMIS individual data elements’ results, there was a decrease in the IQR with each subsequent year from 2015 to 2022 (Figure 2D; Supplemental Table 2).

#### MRRS.

The MRRS average overall aggregate data reporting accuracy increased from 70% in 2015 to 82% in 2022 and remained in the medium-accuracy strata during the assessment period (Figure 3D; Supplemental Table 3). The median overall aggregate data reporting accuracy also increased during the assessment period from 76% in 2015 to 87% in 2022 and improved the accuracy strata from medium to high (Figure 3D; Supplemental Table 3). The IQRs across audit years also gradually decreased (Figure 3D; Supplemental Table 3).

### Health facility accuracy strata.

#### HMIS.

**Figure 4.**
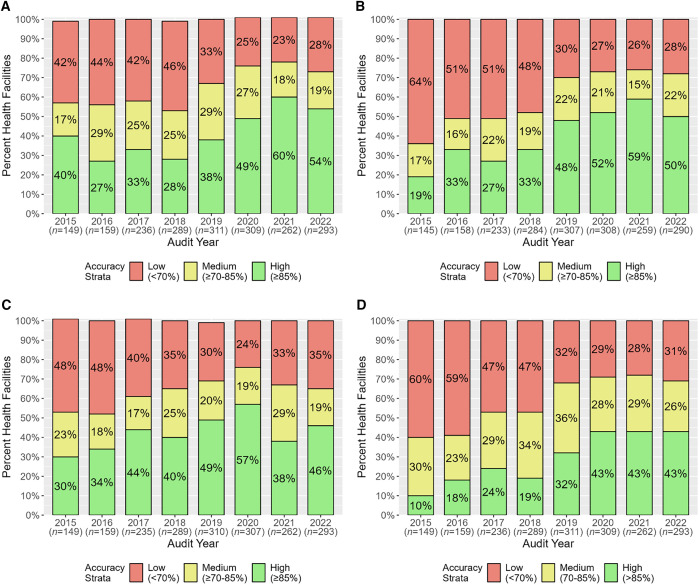
Health facility health management information system aggregate data reporting accuracy by accuracy strata for total outpatient department (OPD) attendance (**A**), rapid diagnostic test (RDT)-tested cases (**B**), total confirmed cases (**C**), and overall (**D**).

The percentage of health facilities with HMIS aggregate data reporting accuracies in the high-accuracy strata for individual data elements and overall increased between 2015 and 2022: from 40% (60/149) to 54% (157/293) for total OPD attendance, from 19% (28/145) to 50% (145/290) for RDT-tested cases, from 30% (44/149) to 46% (135/293) for total confirmed cases, and from 10% (15/149) to 43% (127/293) for overall (Figure 4). In parallel, the percentage of health facilities in the low-accuracy strata gradually decreased during the assessment period for all data elements and overall (Figure 4).

#### MRRS.

**Figure 5.**
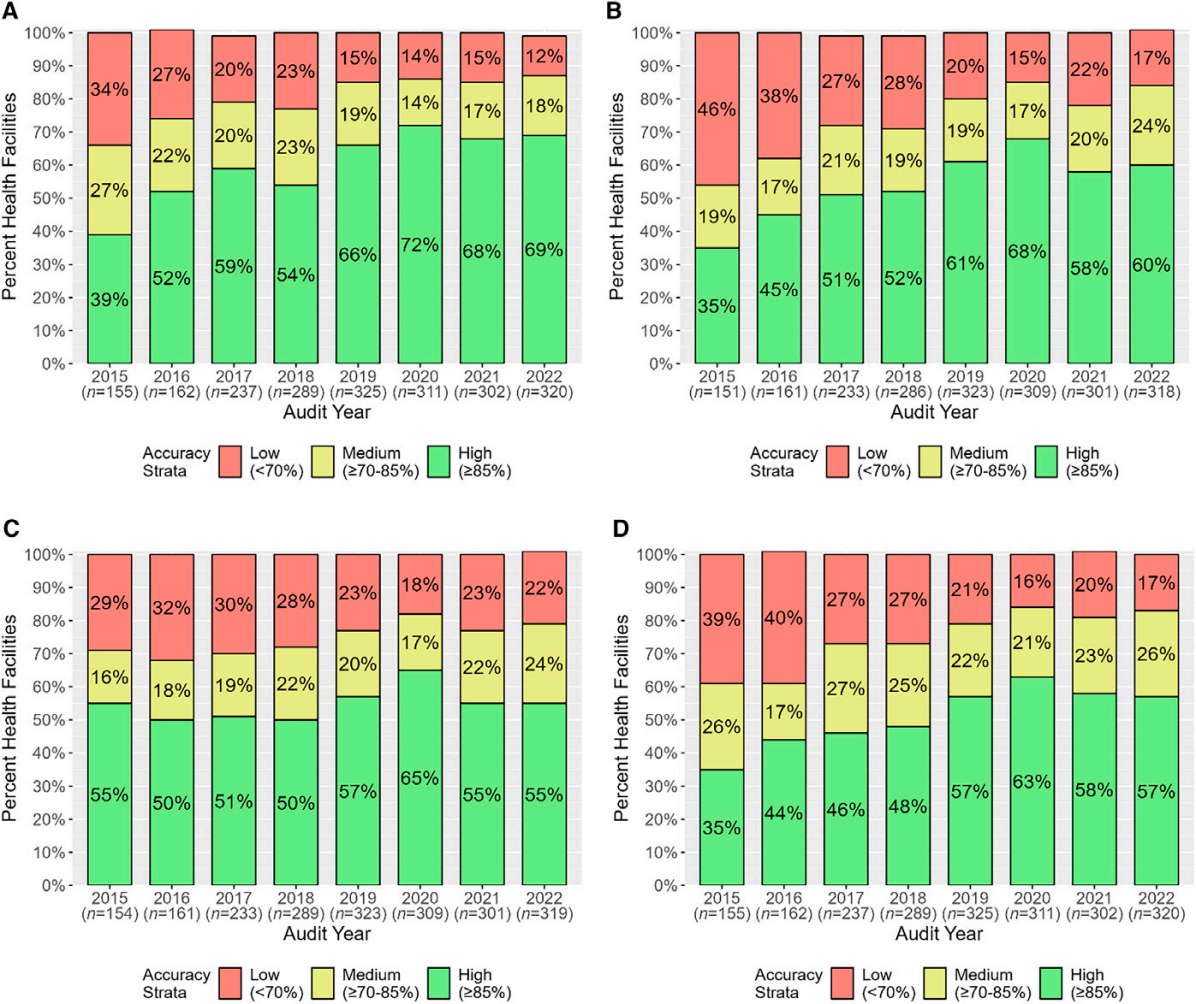
Health facility malaria rapid reporting system aggregate data reporting accuracy by accuracy strata for total outpatient department (OPD) attendance (**A**), rapid diagnostic test (RDT)-tested cases (**B**), RDT-positive cases (**C**), and overall (**D**).

From 2015 to 2022, there was a dramatic increase in the percentage of health facilities in the high-accuracy strata based on MRRS overall aggregate data reporting accuracies for two data elements and overall: from 39% (61/155) to 69% (221/320) for total OPD attendance, from 35% (53/151) to 60% (190/318) for RDT-tested cases, and from 35% (54/155) to 57% (183/320) for overall (Figure 5). These increases were accompanied by substantial decreases in the percentage of health facilities in the low-accuracy strata (Figure 5). In contrast, the percentage of health facilities in the high-accuracy strata based on data reporting accuracies for RDT-positive cases remained the same, 55% in both 2015 (84/154) and 2022 (174/319) (Figure 5).

### Recurrent low-accuracy health facilities.

#### HMIS.

Based on the HMIS DQA results, 12% (79/670) of health facilities were recurrent-low-accuracy health facilities. Generally, both the overall average and median HMIS aggregate data reporting accuracies stayed in the low-accuracy strata from 2015 to 2022, 49% to 63% and 49% to 67%, respectively (Supplemental Figure 4; Supplemental Table 5). There was no clear trend in the IQRs during the assessment period (Supplemental Table 5). Similarly, the percentage of recurrent-low-accuracy health facilities reporting with low accuracy generally stayed the same during the assessment period, with 82% (31/38) in 2015 and 79% (34/43) in 2022 (Supplemental Figure 4).

#### MRRS.

Thirty-one health facilities were considered recurrent-low-accuracy health facilities based on MRRS audits. Both the average and median MRRS overall data reporting accuracies were in the low-accuracy strata across most years, 54% to 71% and 56% to 74%, respectively (Supplemental Table 5; Supplemental Figure 6). The IQR varied year to year across data elements and overall (Supplemental Table 5; Supplemental Figure 6). The percentage of recurrent-low-accuracy health facilities reporting with low overall accuracy decreased from 2015 to 2022, from 92% (11/12) to 44% (8/18) (Supplemental Figure 6). Although there was an overall improvement, the percentage of health facilities in each accuracy strata across years was erratic.

### Number of DQA visits.

#### HMIS.

**Figure 6.**
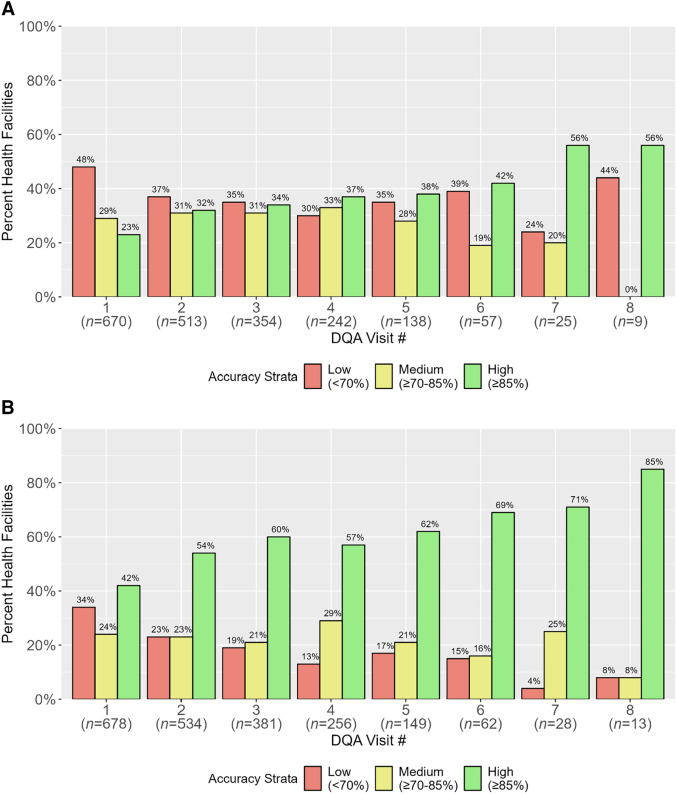
Health facility overall aggregate data reporting accuracy by accuracy strata with each successive health management information system (**A**) and malaria rapid reporting system (**B**) data quality audit (DQA) visit.

Starting at HMIS DQA visit 1, most health facilities reported overall aggregate data reporting accuracies in the low- and medium-accuracy strata, 48% (319/670) and 29%, (196/670), respectively, followed by 23% (155/670) of health facilities in the high-accuracy strata (Figure 6A). From DQA visits 2 to 8, the percentage of health facilities reporting with overall high accuracy increased an average of six percentage points with each successive visit. It was not until DQA visit 7 that over half (56%, 14/25) of health facilities had high overall aggregate data reporting accuracy.

#### MRRS.

Health facility MRRS overall aggregate data reporting accuracies at DQA visit 1 were concentrated mainly in the low- and high-accuracy strata, 34% (229/678) and 42% (284/678), respectively (Figure 6B). The percentage of high-accuracy health facilities from DQA visits 2 to 8 increased an average of five percentage points with each consecutive visit. Over half of facilities (54%, 287/534) reported high overall accuracy as early as DQA visit 2, eventually reaching over 70% by DQA visits 7 (71%, 20/28) and 8 (85%, 11/13).

### Health facility size.

For both HMIS and MRRS, Pearson correlation analysis indicated a weak negative correlation between health facility size (total OPD attendance) and overall aggregate data reporting accuracy (HMIS: *R* = −0.11, *P* = 2 × 10^−6^; MRRS: *R* = −0.063, *P* = 0.0037) (Supplemental Figure 7).

### Malaria case incidence.

#### Health facility-based malaria case incidence.

Using Pearson correlation analysis, there was a weak positive correlation (*R* = 0.15, *P* = 4.4 × 10^−11^) between register-based incidence and HMIS overall aggregate data reporting accuracy, as well as between reported incidence and HMIS overall aggregate data reporting accuracy (*R* = 0.1, *P* = 4.5 × 10^−6^) (Supplemental Figure 8). There was no correlation between register-based incidence and MRRS overall aggregate data reporting accuracy (*R* = 0.011, *P* = 0.61) nor between reported incidence and MRRS overall aggregate data reporting accuracy (*R* = 0.0053, *P* = 0.81) (Supplemental Figure 9).

#### Degree of underreporting and overreporting.

**Figure 7.**
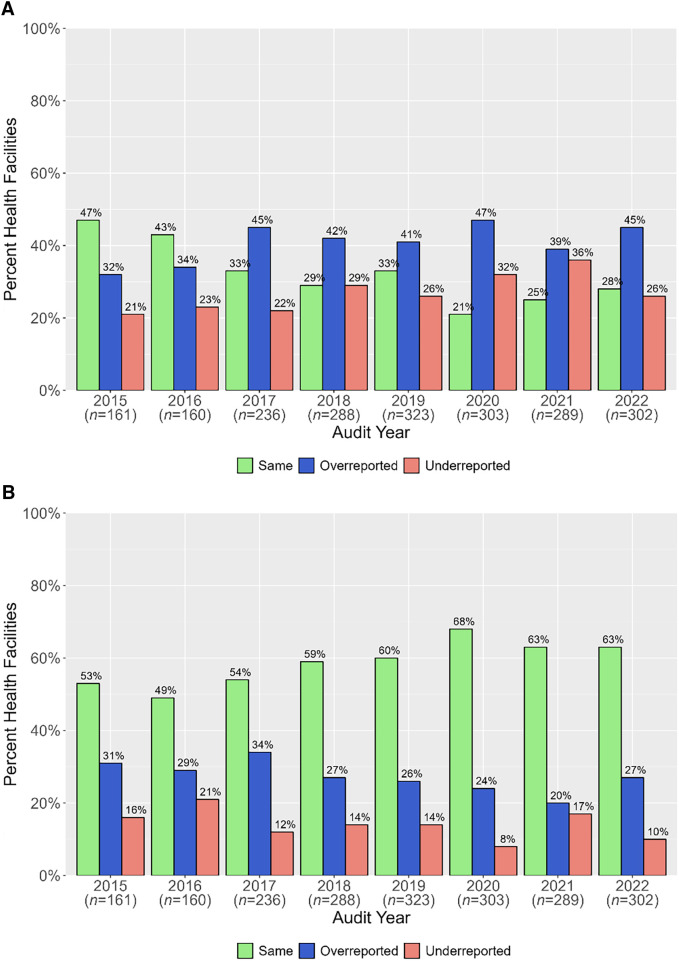
Degree of overreporting and underreporting of reported incidence in comparison with register-based incidence unadjusted (**A**) and adjustment for ±10% allowable error in reported incidence (**B**).

From 2015 to 2022, the percentage of health facilities where the register-based incidence and the reported incidence matched declined from 47% (75/161) in 2015 to 28% (86/302) in 2022 (Figure 7A). There was a corresponding increase in overreported and underreported incidences: from 32% (52/161) in 2015 to 45% (137/302) in 2022 for overreporting and from 21% (34/161) in 2015 to 26% (79/302) in 2022 for underreporting (Figure 7A). When a ±10% reporting error adjustment was applied (Materials and Methods), most reported incidences were the same as their respective register-based incidences across all audit years, increasing from 53% (85/161) in 2015 to 63% (190/302) in 2022 (Figure 7B). The degree of overreported or underreported incidence was less when adjusted and varied between 20% to 34% for overreporting and 8% to 21% for underreporting during the assessment period (Figure 7B).

#### Malaria risk stratification.

**Figure 8.**
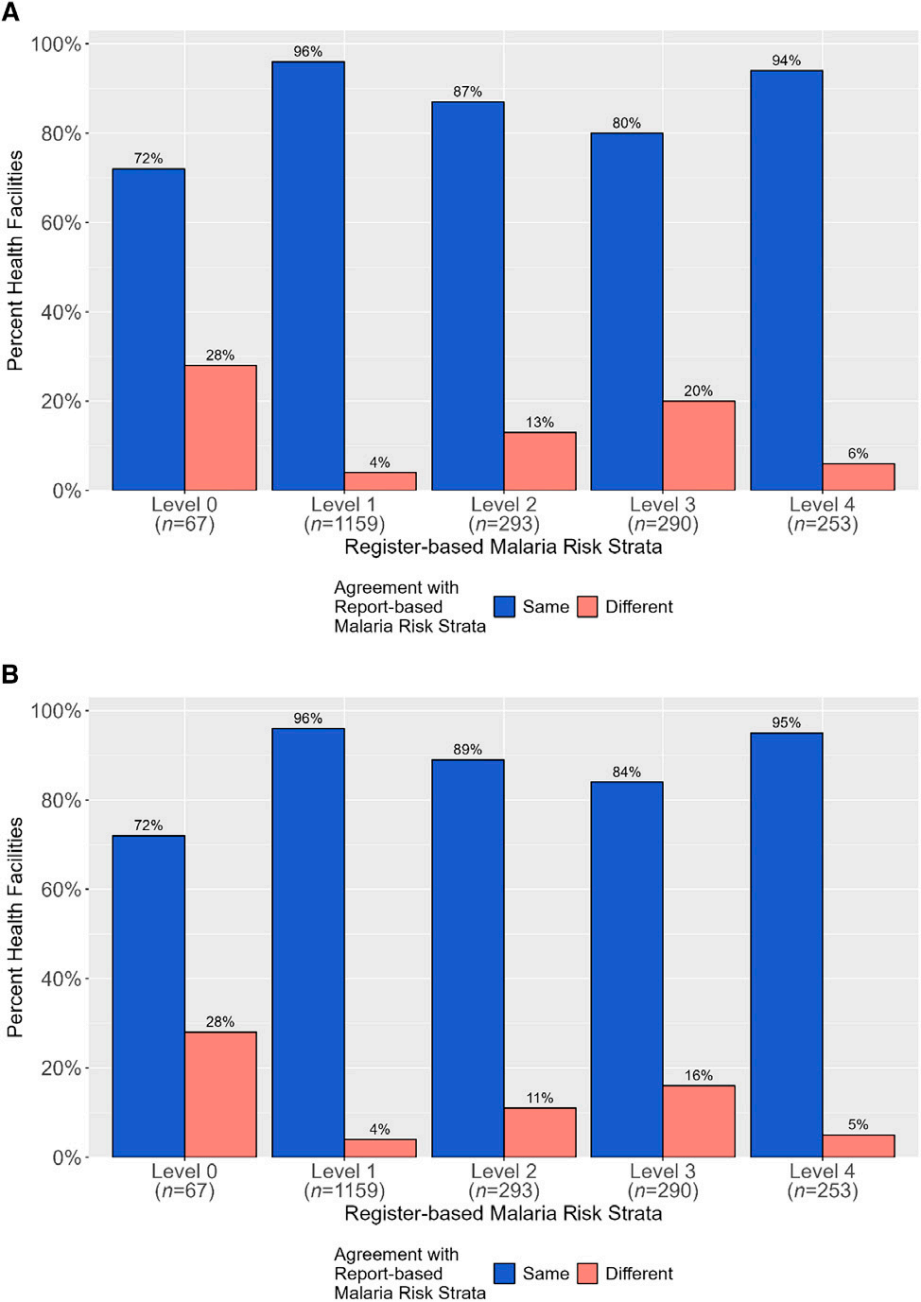
Comparison of malaria risk strata assignment using unadjusted reported incidence (**A**) and reported incidence with adjustment for ±10% allowable error (**B**) to malaria risk strata assignment using register-based incidence.

Over 80% of health facilities assigned to a malaria risk strata based on their reported incidence were also in the same malaria risk strata based on their register-based incidence, particularly at level 1 (“very low malaria”), level 2 (“low malaria”), level 3 (“moderate malaria”), and level 4 (“high malaria”) strata (Figure 8A). When the ±10% error adjustment was applied to reported incidences (Materials and Methods) and used for establishing malaria risk strata, the concordance between report-based risk strata and register-based risk strata was similar to that of the unadjusted results (Figure 8B).

## DISCUSSION

This study showed that malaria aggregate data reporting accuracy improved from 2015 to 2022 in Central, Southern, and Western provinces in Zambia. Both the average and median aggregate data reporting accuracies for total OPD attendance and RDT-tested cases in HMIS and MRRS, total confirmed cases in HMIS, and overall for HMIS and MRRS increased during the assessment period, shifting to a higher accuracy strata by 2022. Similarly, the percentage of high-accuracy health facilities increased for most HMIS and MRRS data elements with a corresponding decrease in the percentage of low-accuracy health facilities. The average and median aggregate data reporting accuracies of RDT positives were generally stable during the assessment period, and there was no change in the percentage of health facilities reporting RDT-tested cases in MRRS with high accuracy. These results suggest that although overall data accuracy improved, a different approach may be needed to further increase the data reporting accuracy of specific data elements, such as RDT positives in MRRS.

The overall improvement in malaria aggregate data reporting accuracy was further complemented by reduction in the IQR over time for all HMIS and MRRS data elements and overall, demonstrating a tighter distribution in the middle 50% of health facility aggregate data reporting accuracies with each consecutive year. In contrast, a subset of health facilities had average and median data reporting accuracies primarily in the low-accuracy strata and had wide IQRs during the assessment period despite receiving multiple DQA visits, suggesting that these facilities tend to remain in the low-accuracy strata for reasons beyond what could be determined in this study. Identifying these recurrent-low-accuracy health facilities could be particularly useful at the district level to prioritize facilities that need additional follow-up to improve data quality. Of note, this assessment evaluated data reporting accuracy using three data elements that are essential for measuring basic malaria indicators like incidence, testing rate, and test positivity rate, as well as for calculating commodity requirements, but there may be additional data elements that are important programmatically and should be considered in future assessments.

When surveillance systems and their shared data elements (total OPD attendance and RDT-tested cases) were compared, the average and median aggregate data reporting accuracies for total OPD attendance and RDT-tested cases in MRRS were consistently greater than those in HMIS each year, even among facilities in lower-accuracy strata. The reason for the difference is unclear because the aggregation of the data, whether monthly (HMIS) or weekly (MRRS), relies on the same registers. Possible reasons for the discrepancy may include completion of HMIS and MRRS reporting forms by different staff, misunderstandings in data element definitions between the two systems, user experience in paper (HMIS) versus electronic (MRRS) reporting, changes in HMIS reporting forms during the assessment period, and inadequate training and/or supervision. There is also a considerable burden on surveillance staff to complete the HMIS reporting form because it encompasses other disease areas; it is possible that surveillance staff experience fatigue and distractions while tallying data that contribute to errors during this monthly activity. Identifying the factors that influence these discrepancies would be valuable for district-level officers in implementation of data quality improvement activities and should be explored in future studies.

With each successive DQA visit (visits 1–8), the percentage of health facilities with high overall aggregate data reporting accuracy (≥85%) increased for both HMIS and MRRS audits. The improvement in health facilities with high overall data reporting accuracy started as early as the second DQA visit for both systems. Although more than half of facilities reported high overall reporting accuracy by DQA visit 2 for MRRS, it took at least seven DQA visits to reach a similar level for HMIS. The difference in HMIS and MRRS accuracies with each consecutive visit suggests that there may be other factors that influence accuracy related to HMIS and should be further explored and addressed. Given the low sample sizes at DQA visits 7 and 8 for both systems, it will be important to monitor these trends as health facilities receive more DQA visits to substantiate the positive influence of repeat DQA visits on reporting accuracy.

Using the HMIS and MRRS register and reported values for total confirmed cases and RDT-positive cases, respectively, more than 40% of health facilities had the same register-based and reported incidences in the first 2 years and then decreased, resulting in most facilities overreporting incidence. A ±10% allowable reporting error in reported incidence resulted in most health facilities having the same register-based and reported incidences each year, suggesting that most reported values were not dramatically different from their respective register values. The impact of overreported and underreported incidence on malaria risk stratification showed that most health facilities would be correctly assigned to a risk strata based on reported incidence compared with register-based incidence, giving a high level of confidence in being able to use reported surveillance data to assign facilities to risk strata. However, there were still some differences observed in assignment, so where RDQAs are implemented, this type of analysis could be used to detect health facilities that have different register-based and report-based malaria risk strata assignments. Particularly for malaria programs whose primary purpose for conducting RDQAs is to support annual risk stratification review, monitor epidemiological trends, and/or determine the impact of interventions, an “allowable” error for health facility-reported incidence and possibly for other metrics should be considered rather than trying to achieve perfect reporting of individual data elements. However, this allowable level of uncertainty depends on the primary use of reported data elements, which must be determined by malaria programs; for example, reporting errors for RDT-tested cases could result in inaccurate forecasting of commodities and negatively impact malaria case management.

Prior to this study, there was an assumption that facilities covering a large population or with high malaria incidence might be introducing more reporting errors because of the large numbers of patient records in registers that must be aggregated for monthly (HMIS) and weekly (MRRS) reporting. Correlation analysis looking at the influence of health facility size or incidence on accuracy showed there was no apparent effect. Accordingly, there are likely other contextual factors that impact malaria data reporting accuracy at both the district and health facility levels, including staff turnover, frequency of training, and supervision. Staff turnover can result in loss of institutional knowledge and increased workload for other staff, and inadequate training and supervision can lead to substandard malaria surveillance practices and limited feedback, all of which negatively impact data quality. This assessment was unable to explore these factors with the available DQA data; as a result, it may be useful to include indicators in addition to the current DQA tool that can capture these factors, such as new staff hired during the DQA audit period, number of supervision visits, and surveillance training received in the previous year. This would support future programmatic evaluations or other studies describing data reporting accuracy trends in Zambia to identify key challenges and develop effective data quality improvement activities.

Evaluating overall aggregate data reporting accuracy using RDQA results can inform DQA best practices tailored to country needs. Understanding how different health levels use routine DQA results can form the foundation for a series of use cases and actions that guide malaria programs. In countries where there is significant donor investment in RDQAs, a systematic approach to measuring trends in aggregate malaria data reporting accuracy can rapidly assess its effectiveness across audited health facilities. Provincial and district level teams may also benefit from user-friendly visualizations such as those produced in this assessment, except modified for each province or district; these visualizations could be used to rapidly review RDQA performance indicators, track progress, and identify and respond to issues at health facilities. At the district level, selection of health facilities for RDQAs may not be clear nor followed, and previous DQA performance may not be examined; as a result, district- and health facility-specific analysis of temporal trends in malaria data reporting accuracy combined with other data quality attributes (reporting rate, completeness, timeliness, validity, consistency) could be used to develop criteria that optimize selection and sampling of health facilities, particularly low-accuracy or recurrent-low-accuracy health facilities, for the next DQA. This targeted approach may accelerate improvements in HMIS and MRRS aggregate data reporting accuracy, potentially reducing the number of audits required for facilities to reach high accuracy. Examining aggregate data reporting accuracy of certain indicators using DQA data could also support annual risk stratification reviews at the national level by identifying and addressing health facilities that were incorrectly assigned to risk strata, which has implications for program planning and resource allocation.

Across all health levels, significant human and financial resources are required for RDQAs, which can limit its scope; hence, evaluating trends in malaria data reporting accuracy in audited health facilities can provide insight into ways to simplify the DQA process, such as reducing the number of data elements reviewed or auditing fewer weeks to facilitate scale-up of coverage; however, the cost-effectiveness, time gained, and additional health facilities audited would need to be measured before modification of current DQA practices. Lastly, because data quality issues are not siloed to only one disease program, a holistic and tailored approach to tracking aggregate data reporting accuracy across other disease programs conducting RDQAs should be explored.

## CONCLUSION

From 2015 to 2022, malaria aggregate data reporting accuracy improved in health facilities that received RDQAs in Central, Western, and Southern provinces in Zambia. With each successive DQA, overall aggregate data reporting accuracy increased with most health facilities in the medium- or high-accuracy strata, indicating that RDQAs should be maintained to continue these positive trends in data reporting accuracy. This study presents a standardized approach to evaluating temporal trends in malaria aggregate data reporting accuracy that can be used at all health levels for a range of decisions, from rapid identification of low-accuracy health facilities by the district level to annual risk stratification at the national level. Country programs should consider routine analysis of DQA data over time to evaluate changes in data quality indicators and establish DQA best practices tailored to program priorities.

## Supplemental Materials

10.4269/ajtmh.24-0429 Supplemental Materials
