# Supplementary material for: Documenting Trends in Malaria Data Reporting Accuracy Using Routine Data Quality Audits in Zambia, 2015–2022
Source: Am J Trop Med Hyg. 2024 Nov 26;112(2):274–85. doi: 10.4269/ajtmh.24-0429 (PMC11803651; doi:10.4269/ajtmh.24-0429)

S1. Malaria surveillance data flow process at health facility and community levels for a) HMIS and b) MRRS.

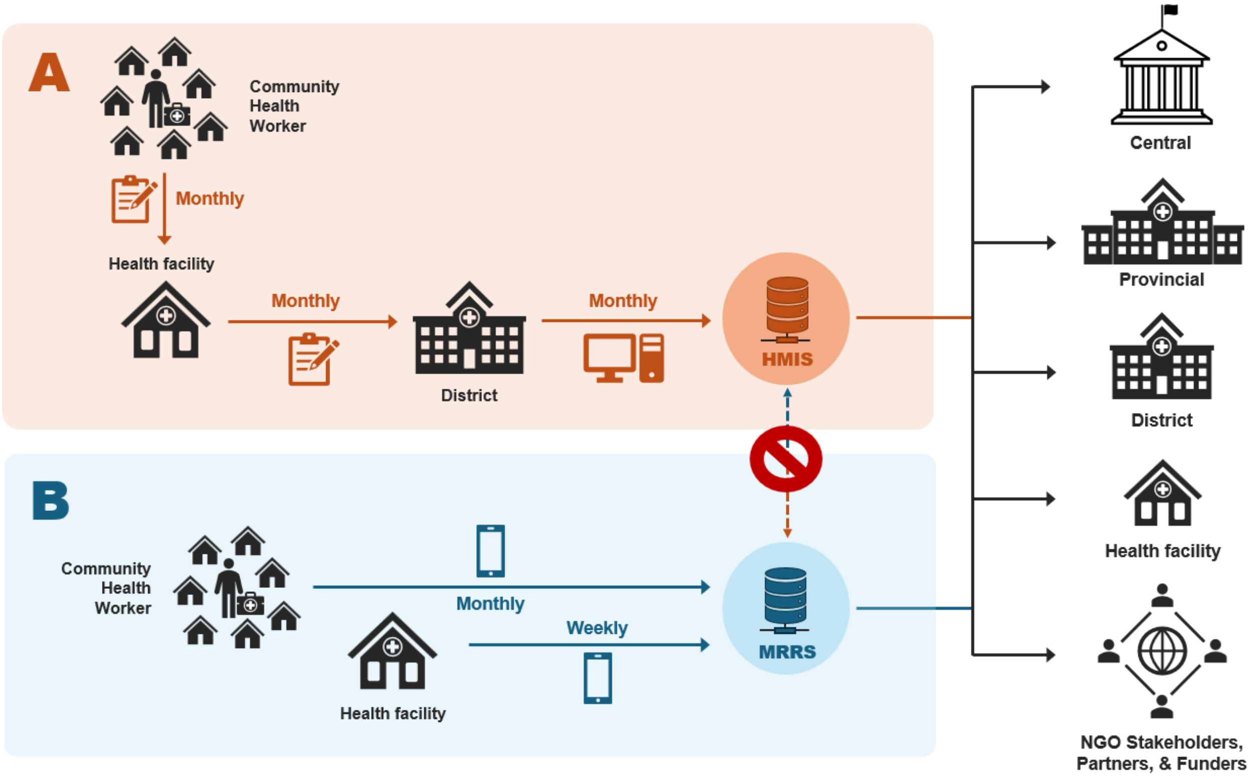

S2. Descriptive statistics for boxplots of HMIS aggregate data reporting accuracy in for a) total OPD attendance, b) RDT tested cases, c) total confirmed cases, and d) overall

a) Total OPD attendance

Audit Year	Average Aggregate Data Reporting Accuracy	Median Aggregate Data Reporting Accuracy	Interquartile Range
2015	65%	76%	39
2016	62%	73%	42
2017	70%	76%	33
2018	65%	74%	37
2019	73%	79%	31
2020	77%	84%	23
2021	80%	89%	22
2022	78%	87%	26

b) RDT tested cases

Audit Year	Average Aggregate Data Reporting Accuracy	Median Aggregate Data Reporting Accuracy	Interquartile Range
2015	52%	58%	51
2016	59%	69%	58
2017	60%	68%	46
2018	60%	72%	49
2019	74%	84%	32
2020	75%	85%	27
2021	77%	88%	28
2022	75%	85%	27

c) Total confirmed cases

Audit Year	Average Aggregate Data Reporting Accuracy	Median Aggregate Data Reporting Accuracy	Interquartile Range
2015	61%	72%	49
2016	62%	71%	54
2017	66%	82%	46
2018	71%	79%	29
2019	74%	84%	29
2020	77%	87%	24
2021	74%	78%	29
2022	71%	82%	36

d) Overall

Audit Year	Average Aggregate Data Reporting Accuracy	Median Aggregate Data Reporting Accuracy	Interquartile Range
2015	59%	64%	34
2016	61%	63%	33
2017	66%	72%	34
2018	66%	72%	29
2019	73%	78%	22
2020	76%	82%	23
2021	77%	81%	23
2022	75%	80%	26

S3. Descriptive statistics for boxplots of MRRS aggregate data reporting accuracy in for a) total OPD attendance, b) RDT tested cases, c) RDT positive cases, and d) overall

a) Total OPD attendance

Audit Year	Average Aggregate Data Reporting Accuracy	Median Aggregate Data Reporting Accuracy	Interquartile Range
2015	70%	80%	33
2016	75%	86%	25
2017	80%	88%	23
2018	77%	86%	24
2019	84%	91%	15
2020	84%	92%	13
2021	82%	91%	15
2022	85%	90%	14

b) RDT tested cases

Audit Year	Average Aggregate Data Reporting Accuracy	Median Aggregate Data Reporting Accuracy	Interquartile Range
2015	64%	74%	44
2016	67%	80%	49
2017	76%	85%	26
2018	75%	86%	29
2019	80%	88%	19
2020	83%	91%	14
2021	79%	89%	22
2022	82%	88%	16

c) RDT positive cases

Audit Year	Average Aggregate Data Reporting Accuracy	Median Aggregate Data Reporting Accuracy	Interquartile Range
2015	76%	88%	31
2016	73%	85%	39
2017	75%	85%	28
2018	77%	85%	28
2019	79%	88%	24
2020	82%	90%	17
2021	77%	87%	23
2022	79%	87%	24

d) Overall

Audit Year	Average Aggregate Data Reporting Accuracy	Median Aggregate Data Reporting Accuracy	Interquartile Range
2015	70%	76%	34
2016	72%	80%	36
2017	77%	83%	25
2018	76%	83%	26
2019	81%	88%	19
2020	83%	90%	16
2021	80%	88%	21
2022	82%	87%	16

S4. HMIS recurrent low accuracy health facilities a) overall aggregate data reporting accuracy and b) accuracy strata

a) Overall aggregate data reporting accuracy

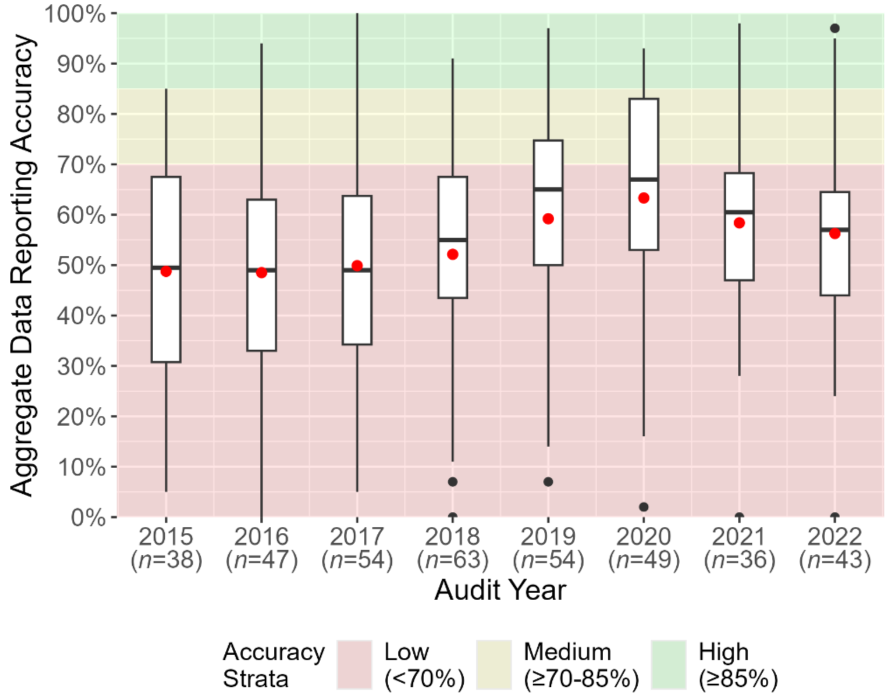

b) Health facilities in each accuracy strata

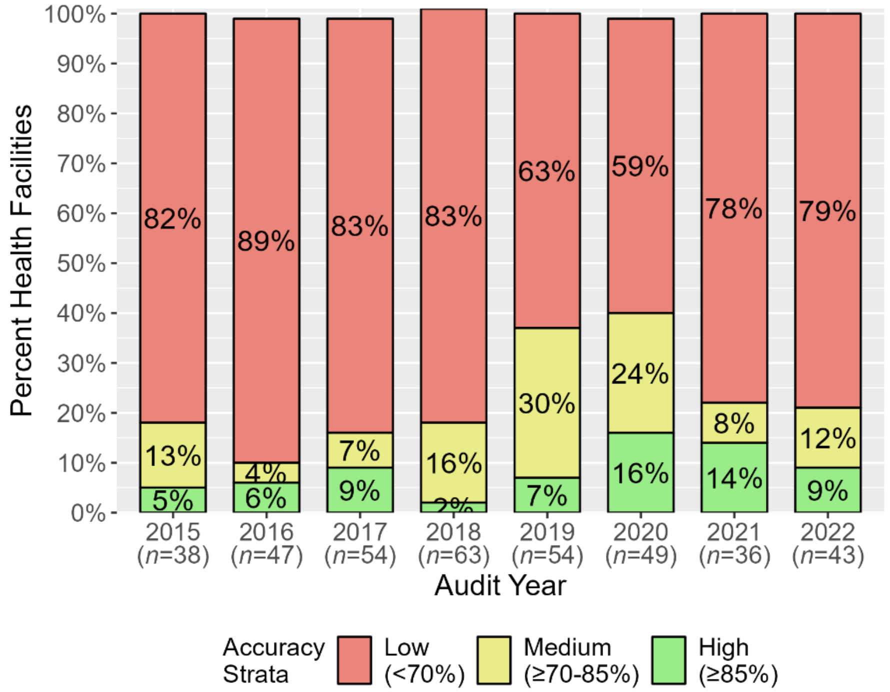

S5. Descriptive statistics for boxplots of a) HMIS and b) MRRS overall aggregate data reporting accuracy at recurrent low accuracy health facilities

a) HMIS

Audit Year	Average Aggregate Data Reporting Accuracy	Median Aggregate Data Reporting Accuracy	Interquartile Range
2015	49%	50%	37
2016	49%	49%	30
2017	50%	49%	30
2018	52%	55%	24
2019	59%	65%	25
2020	63%	67%	30
2021	58%	61%	21
2022	56%	57%	21

b) MRRS

Audit Year	Average Aggregate Data Reporting Accuracy	Median Aggregate Data Reporting Accuracy	Interquartile Range
2015	54%	60%	17
2016	55%	56%	20
2017	58%	60%	11
2018	62%	66%	31
2019	55%	57%	22
2020	58%	57%	25
2021	69%	72%	25
2022	71%	74%	33

S6. Malaria rapid reporting system recurrent low accuracy health facilities by a) overall aggregate data reporting accuracy and b) accuracy strata.

a) Overall aggregate data reporting accuracy

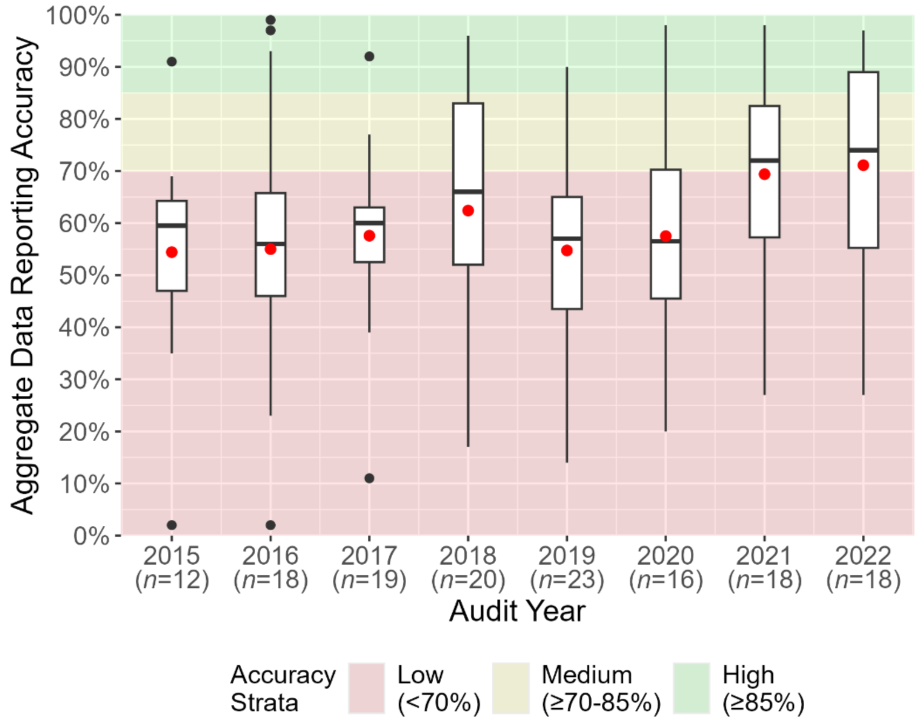

b) Health facilities in each strata

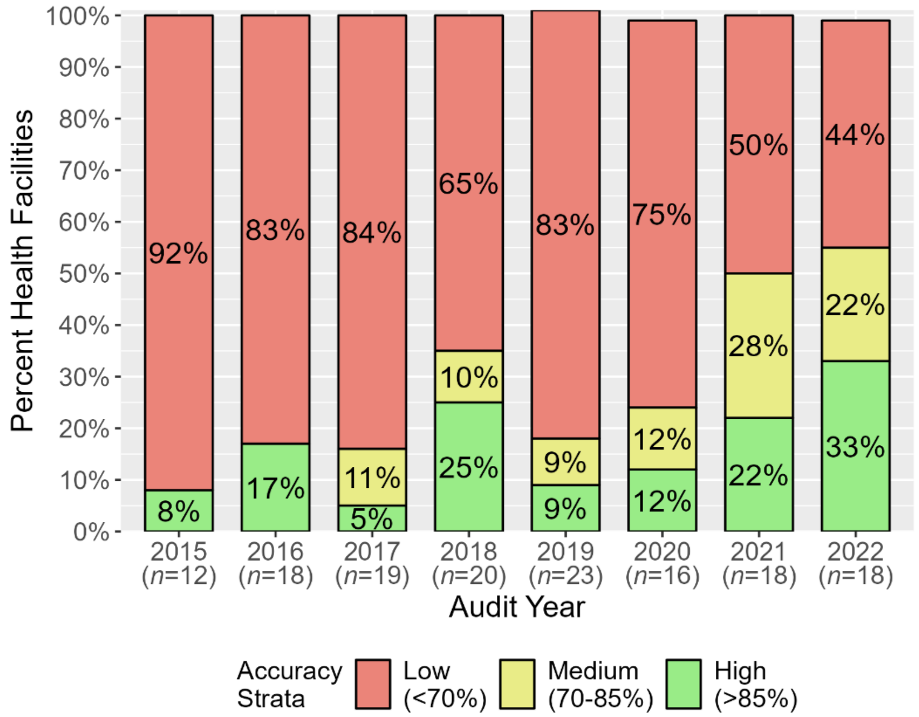

S7. Pearson correlation analysis of health facility size and overall aggregate data reporting accuracy based on a) HMIS and b) MRRS routine DQAs

a) HMIS

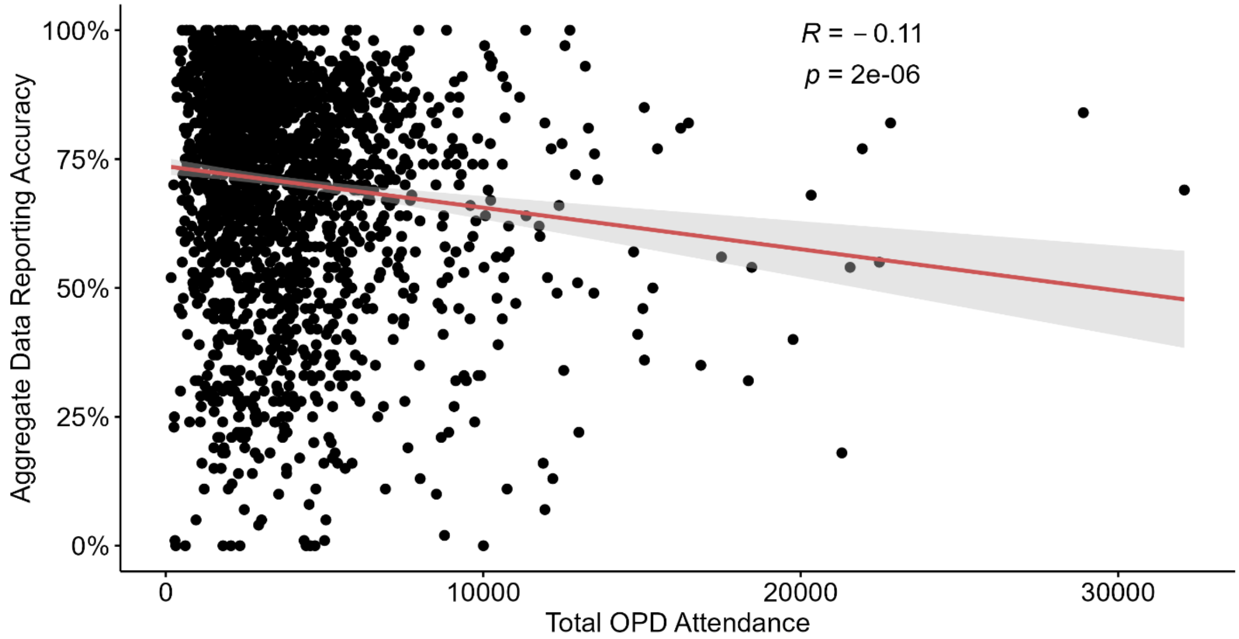

b) MRRS

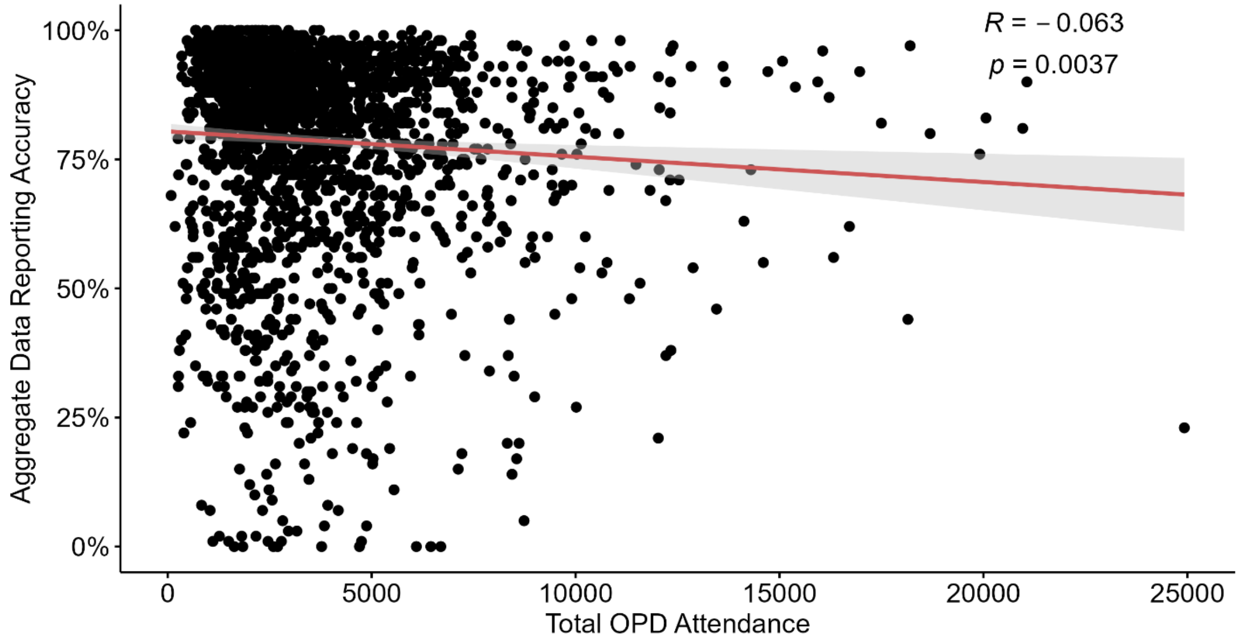

S8. Pearson correlation analysis of health facility a) register-based and b) reported incidence and HMIS overall aggregate data reporting accuracy for each six-month audit period

a) Register-based incidence

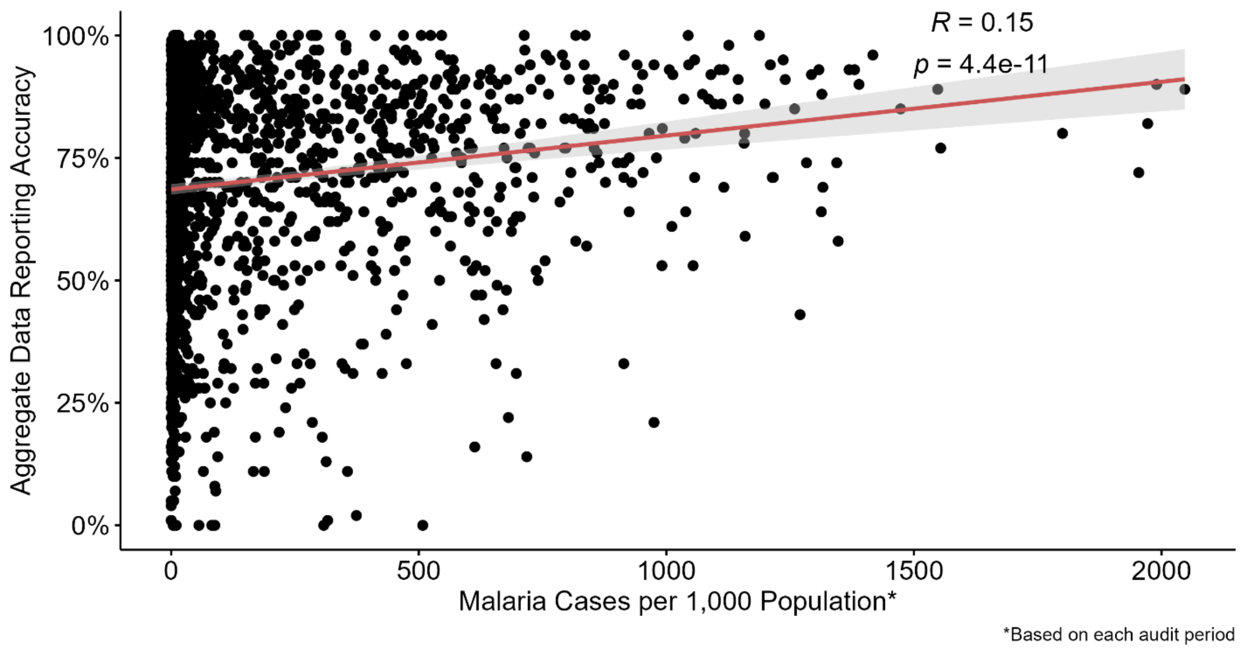

b) Reported incidence

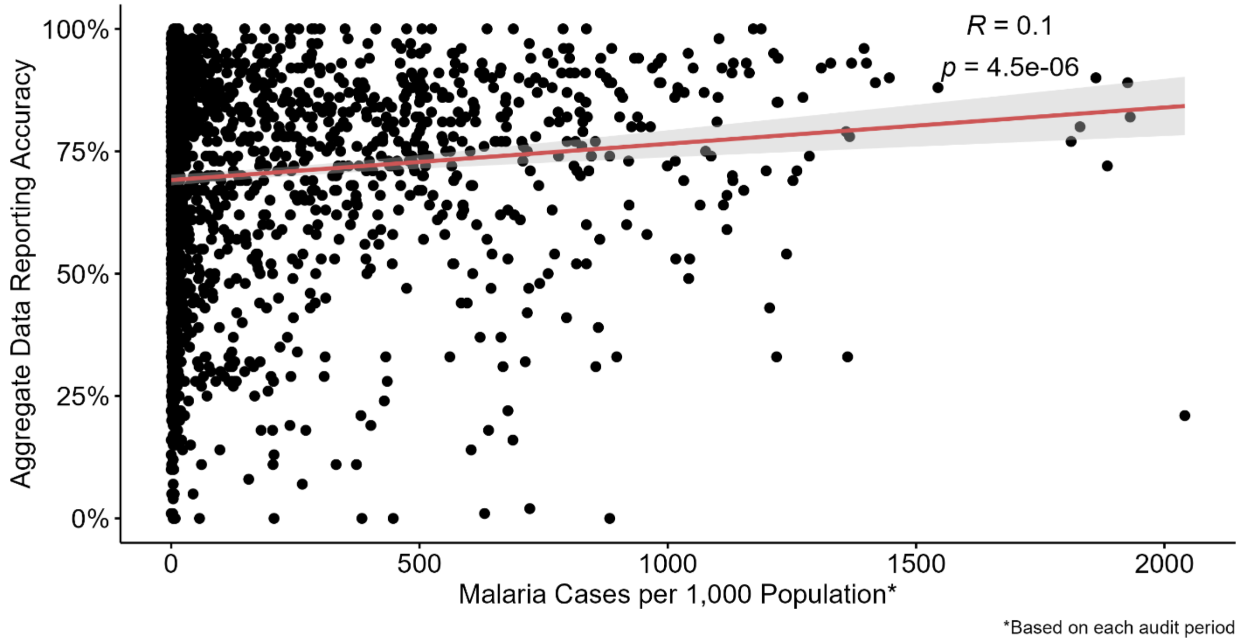

S9. Pearson correlation analysis of health facility a) register-based and b) reported incidence and MRRS overall aggregate data reporting accuracy for each six-month audit period

a) Register-based incidence

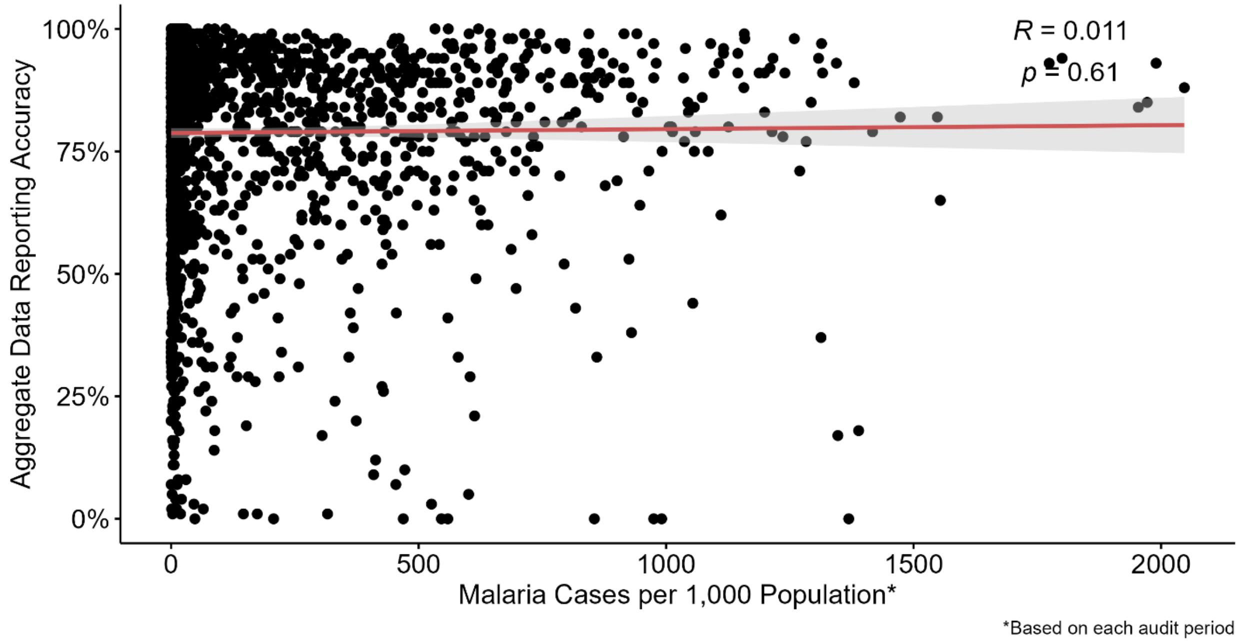

b) Reported incidence

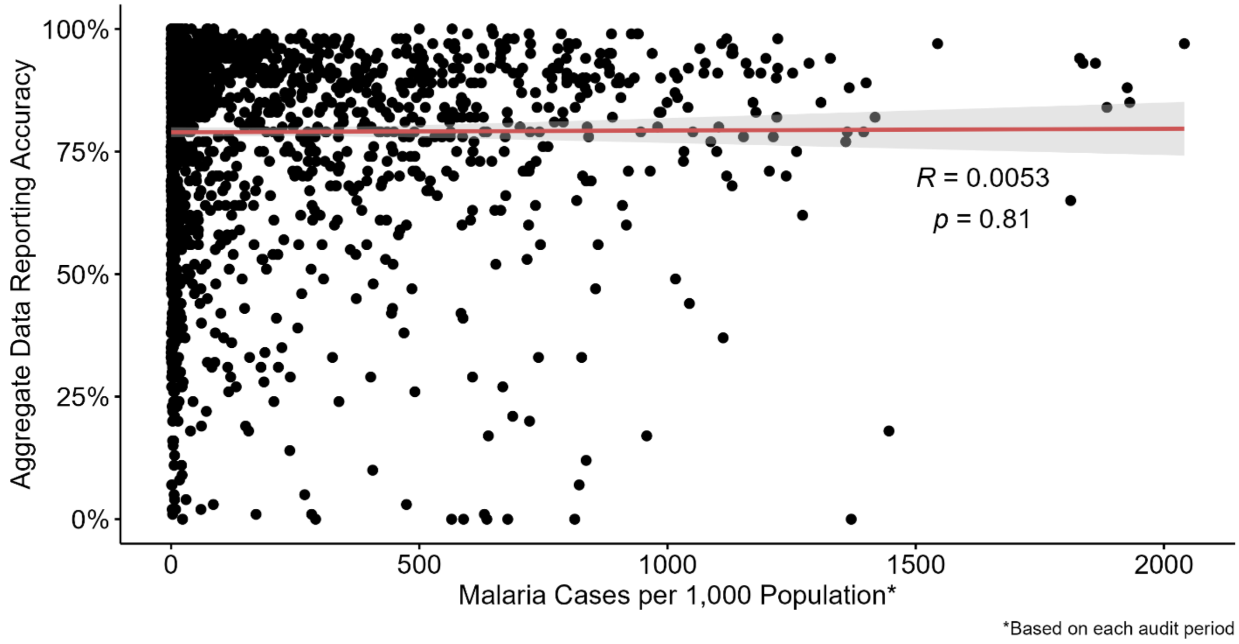

Supplement: Supplemental Materials [file tpmd240429.SD1.pdf]
